# Factors Influencing the Loss of Ambulation in Patients With Amyotrophic Lateral Sclerosis: A Retrospective Cohort Study

**DOI:** 10.1002/hsr2.71282

**Published:** 2025-09-22

**Authors:** Takahiro Miyashita, Eiki Tsushima, Hirofumi Ogihara

**Affiliations:** ^1^ Department of Physical Therapy Saku Central Hospital Nagano Japan; ^2^ Graduate School of Health Sciences Hirosaki University Aomori Japan; ^3^ Division of Physical Therapy, Department of Rehabilitation, Faculty of Health Sciences Nagano University of Health and Medicine Nagano Japan

**Keywords:** amyotrophic lateral sclerosis, Cox regression, diabetes mellitus, duration to diagnosis, loss of ambulation

## Abstract

**Background and Aims:**

Loss of ambulation in patients with amyotrophic lateral sclerosis (ALS) reduces their social participation and quality of life. Moreover, loss of ambulation is one of the biggest obstacles for patients and their caretakers. However, prognostic factors for loss of ambulation in patients with ALS have not been clarified. This study aimed to investigate the time to loss of ambulation in Japanese patients with ALS and to identify factors associated with loss of ambulation.

**Methods:**

Overall, 62 patients with ALS were enrolled. Survey items included patient background, pre‐existing medical conditions, and laboratory data. Cox proportional hazards regression analysis was performed with the time to loss of ambulation as the dependent variable and age at onset, sex, onset type, duration to diagnosis, ALS severity, diabetes mellitus (DM), and %forced vital capacity (%FVC) as independent variables.

**Results:**

The median time to loss of ambulation for all ALS patients was 16.5 (10.0–31.3) months. Cox regression analysis revealed a significant association between duration to diagnosis (HR: 0.94, 95% CI: 0.91–0.97, *p* < 0.001) and DM (HR: 3.30, 95% CI: 1.62–6.69, *p* < 0.001).

**Conclusions:**

Time to diagnosis and history of diabetes are important factors associated with the time to loss of ambulation in patients with ALS.

**Trial Registration:**

Retrospectively registered.

## 1 Introduction

Amyotrophic lateral sclerosis (ALS) is a rare and complex neurodegenerative disease characterized by rapid progression of weakness and loss of motor function [1]. Patients with ALS survive 2–5 years after the disease onset, leading to death or severe disability [2]. In addition to age, bulbar onset, late diagnosis, rate of disease progression, and noninvasive positive pressure ventilation therapy [3, 4, 5, 6], ambulation is an important predictor of prognosis in patients with ALS [7, 8]. In neurological diseases, including ALS, the impairments in ambulation reduce social participation and quality of life and are among the most considerable obstacles experienced by the patients and their caregivers [9].

Previous studies have reported that lower‐limb muscle strength and fatigue are associated with reduced ambulation in patients with ALS [10, 11]. However, these are all cross‐sectional analyzes and do not focus on the period of loss of ambulation [10, 11]. If the risk factors for ambulation prognosis are identified at the time of diagnosis, clinicians may be able to apply them clinically as useful information for treatment planning and goal setting.

Nevertheless, reports on the duration to loss of ambulation in patients with ALS are lacking, and the prognostic determinants for ambulatory capacity remain incompletely elucidated. The present study investigated the time to loss of ambulation in Japanese patients with ALS and to identify the factors associated with loss of ambulation.

## 2 Methods

This was a retrospective cohort study.

### 2.1 Patients

**Figure 1:**
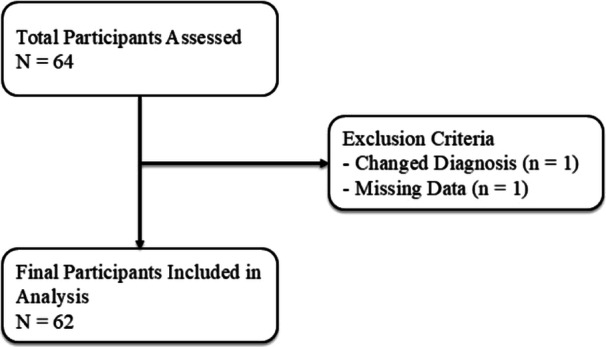
Flowchart for the enrollment of the study participants.

All patients were recruited from the Department of Neurology, Saku Central Hospital, Nagano, Japan. The patients selected for this study met the following criteria: (1) patients with a confirmed diagnosis of ALS according to the revised El Escorial criteria or Awaji criteria between May 2007 and January 2022 [12, 13] and (2) patients who underwent rehabilitation during hospitalization. Patients were excluded if (1) they disagreed with the use of data from this study; and (2) they had a diagnosis of ALS but the diagnosis was changed during the study course. Finally, 62 patients with ALS were selected for analysis (Figure 1). The point of onset was characterized as the time when the patients initially became aware of their muscular debility, swallowing difficulties, speech impediments, and respiratory challenges. Meanwhile, loss of ambulation was defined as the patient's inability to walk without assistance, specifically indicating dependence on a wheelchair for both indoor and outdoor mobility [14].

### 2.2 Data Collection

Patient data were collected at admission. The participants' characteristics, including age at ALS onset, sex, site of onset, duration to diagnosis, grip strength, body mass index (BMI), smoking and alcohol history, ALS severity, occupational history, and number of days to loss of ambulation, were collected from electronic records. Furthermore, the site of ALS onset was classified into *bulbar onset* and *spinal onset* based on the initial symptoms [15, 16]. Grip strength was measured twice on each side, with the average value used in the analysis. BMI was calculated using the following formula: weight (kg)/[height (m)]². The severity of ALS was classified into five levels according to the Ministry of Health, Labor and Welfare classification [17, 18]: grade 1, generally able to perform household chores and work; grade 2, the patient has difficulty with performing household chores and with employment but is generally independent in performing activities of daily living (personal care); grade 3, the patient is unable to eat, urinate, or move around on their own, and requires assistance in daily living; grade 4, the patient has difficulty breathing, expectorating, or swallowing; grade 5, the patient has a tracheostomy, receives parenteral nutrition (tube feeding, central venous nutrition, etc.), or uses a ventilator. The higher the number, the more severe the illness, and patients with a grade ≥ 2 are eligible for medical expense subsidies for designated intractable diseases. Furthermore, occupations were classified into *white‐collar* and *blue‐collar* categories based on the Japanese Standard Classification of Occupations (12 categories) by the Japanese Ministry of Internal Affairs and Communications. *White‐collar* occupations were defined as managerial, professional, and technical clerical and sales occupations. The *blue‐collar* category included service, security, agriculture, forestry, fishery and production process workers, transportation and machine operators, construction and mining workers and transportation, and cleaning and packaging workers [19, 20, 21].

Disease history included orthopedic, cerebrovascular, and cardiovascular diseases, diabetes mellitus (DM), dementia, and malignancy. The examination data included blood gas data, including partial pressure of arterial oxygen (PaO_2_), partial pressure of arterial carbon dioxide (PaCO_2_), and hydrogen carbonate (HCO_3_^−^). Pulmonary function was examined as %forced vital capacity (%FVC) and %predicted forced expiratory volume in 1 s (%FEV1.0). Blood gas tests were performed by neurologists mainly by puncturing the femoral artery, and the pulmonary function tests were performed by clinical laboratory technicians by spirometry.

### 2.3 Statistical Analysis

Descriptive statistics are presented for patient background, history and laboratory data. Continuous variables with a normal distribution are presented as mean ± standard deviation. In cases where normality is not ascertained, data are expressed median along with the interquartile range. The assessment of data normality ensued through the examination of distribution patterns using histograms and validation using the Shapiro–Wilk test. As an exploratory step, Spearman's rank correlation coefficients were calculated to assess the associations between clinical variables and time to loss of ambulation. Subsequently, a Cox proportional hazards regression model was used to identify the factors associated with loss of ambulation. The proportional hazards assumption was tested using Schoenfeld residuals, and no violations were detected (global test *p* = 0.313). The independent variables included in the multivariable model were: Age at onset, Sex, Onset type, Duration to diagnosis, ALS severity, Diabetes mellitus, and %FVC. These variables were selected based on clinical relevance and prior literature. Demographic factors (age, sex), clinical features (Onset type, ALS severity, and Duration to diagnosis), and respiratory function (%FVC) have all been implicated in previous studies as potential predictors of ALS prognosis or functional decline [3, 6, 17, 22, 23]. In addition, diabetes mellitus was included due to recent findings suggesting its potential impact on ALS progression and functional outcomes [24]. For visualization purposes, Kaplan–Meier survival curves were constructed for the variables that were found to be statistically significant in the Cox regression analysis. The log‐rank test was used to compare the time to loss of ambulation between groups. The significance level was set at 5% (two‐sided). All statistical analyzes were performed using SPSS ver. 28.0 (IBM Corporation; Armonk, New York, US). Details of the variable coding are provided in Supporting Information Table 1.

This study was conducted in accordance with the STROBE (Strengthening the Reporting of Observational Studies in Epidemiology) guidelines for reporting observational studies (Supporting Information Table 2).

### 2.4 Ethical Considerations

This study was approved by the Ethics Committee of Graduate School of Medicine, Hirosaki University (Approval No. 2021‐002) and the Institutional Review Board of JA Nagano Koseiren Saku Central Hospital Clinical Research and Trial Center (Approval No. 202005–07). The requirement for individual informed consent was waived by both committees because of the retrospective and observational nature of the study and the use of anonymized data. This waiver complies with the Declaration of Helsinki and the ethical guidelines for medical and health research involving human subjects issued by the Ministry of Health, Labor and Welfare of Japan. An opt‐out procedure was performed, and study information was publicly disclosed on the hospital's bulletin board. All methods were carried out in accordance with relevant guidelines and regulations.

## 3 Results

The patient background, underlying disease and laboratory data are shown in Table 1. No patient had familial ALS. There were 41 male and 21 female patients. Bulbar onset and spinal onset occurred in 19 and 43 patients, respectively. Among the 43 patients with spinal onset, 27 had upper limb onset, 14 had lower limb onset, and 2 had trunk onset. Orthopedic disease accounted for half of the patients (29 patients, 47.5%), followed by cardiovascular disease (14 patients, 23%), DM (13 patients, 21.3%), and cerebrovascular disease (12 patients, 19.7%). Only %FVC (reference value: 80%) and PaO_2_ (reference value: 80 millimeters of mercury) were below the reference values. All patients lost ambulation at 10.0–31.3 (median = 16.5) months. Bulbar onset occurred at a median of 20.0 (8.3–31.5) months, and spinal onset occurred at 16.5 (10.3–33.3) months; however, the difference was not statistically significant (*p* = 0.89).

**Table 1:** Clinical characteristics of the participants with amyotrophic lateral sclerosis.

Basics		
Age at onset	(years old)	67.0 ± 10.2
Sex	(male/female)	41/21
Onset type	(bulbar/spinal)	19/43
Duration to diagnosis	(months)	11.0 (6.8–17.5)
Grip	(kg)	16.5 ± 7.4
BMI	(kg/m^2^)	21.5 ± 4
ALS severity	(1/2/3/4/5)	13/18/9/20/2
Occupation	(blue‐collar/white‐collar)	37/14
Ever smoked	[*n* (%)]	33 (53.2)
Ever drank	[*n* (%)]	31 (50.0)
Medical history		
Musculoskeletal disease	[*n* (%)]	29 (47.5)
Cerebrovascular disease	[*n* (%)]	12 (19.7)
Cardiovascular diseases	[*n* (%)]	14 (23.0)
Respiratory diseases	[*n* (%)]	5 (8.2)
Diabetes mellitus	[*n* (%)]	13 (21.3)
Kidney disease	[*n* (%)]	5 (8.2)
Malignant neoplasms	[*n* (%)]	6 (9.8)
Autoimmune disease	[*n* (%)]	4 (6.6)
Dementia	[*n* (%)]	0
Clinical data		
%FVC	(%)	76.2 (55.9–103.3)
%FEV1.0	(%)	93.2 (79.1–103.9)
PaO_2_	(mmHg)	77.5 (68.6–87.8)
PaCO_2_	(mmHg)	39.1 (35.9–45.6)
HCO^3−^	(mEq/L)	26.2 (23.4–28.8)

*Note:* Data are expressed as mean ± standard deviation or median (interquartile range). Occupation: 37 blue‐collar and 14 white‐collar workers; the remaining 11 patients were retired or not employed.

Abbreviations: %FVC: percent predicted forced vital capacity, %FEV1.0: percent predicted forced expiratory volume in 1 s, PaO_2_: partial pressure of arterial oxygen, PaCO_2_: arterial partial pressure of carbon dioxide, HCO^3−^: hydrogen carbonate.

The results of the correlation analysis showed that the time to loss of ambulation was correlated with age at onset (*r* = − 0.42, *p* < 0.01), duration to diagnosis (*r* = 0.62, *p* < 0.01), ALS severity (*r* = − 0.34, *p* < 0.05), DM (*r* = − 0.35, *p* < 0.05), and %FVC (*r* = 0.40, *p* < 0.05) (Table 2).

**Table 2:** Correlation between time to loss of ambulation and variables.

Variables	Duration to loss of ambulation	
	*r*	*p* value
Age at onset	−0.423**	0.005
Sex	−0.02	0.89
Occupation	0.06	0.72
Onset type	0.02	0.90
Duration to diagnosis	0.615**	< 0.001
ALS severity	−0.357*	0.02
Musculoskeletal diseases	0.01	0.93
Cerebrovascular diseases	−0.07	0.64
Cardiovascular diseases	−0.01	0.97
Respiratory diseases	−0.16	0.30
Diabetes mellitus	−0.352*	0.02
Kidney diseases	−0.21	0.18
Autoimmune diseases	−0.15	0.34
Malignant neoplasms	0.00	0.98
Grip	0.11	0.53
BMI	0.22	0.17
%FVC	0.393*	0.03
FEV1.0%	0.35	0.05
PaCO_2_	−0.08	0.64
PaO_2_	−0.08	0.67
HCO^3−^	−0.33	0.07
Smoking	−0.16	0.32
Drinking	0.17	0.29

* Correlation coefficient is significant at the 5% level (two‐sided).

** Correlation coefficient is significant at the 1% level (two‐sided).

**Figure 2:**
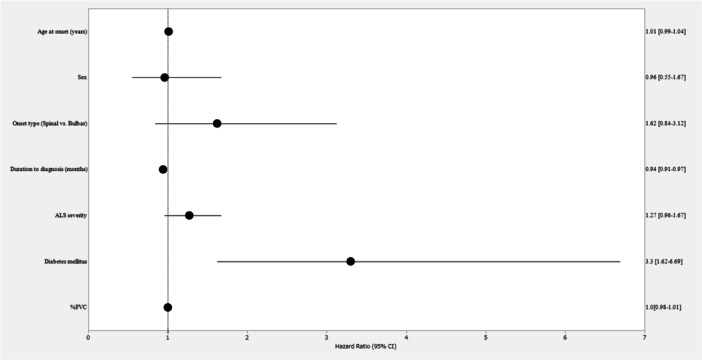
Forest plot of multivariable Cox regression analysis for time to loss of ambulation. Forest plot of multivariable Cox regression analysis showing adjusted hazard ratios (HRs) and 95% confidence intervals (CIs) for factors associated with time to loss of ambulation in patients with ALS. Reference categories: bulbar onset and absence of diabetes mellitus.

**Figure 3:**
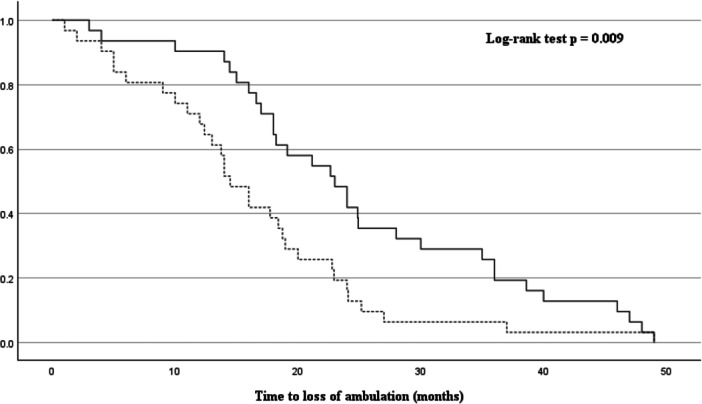
Kaplan–Meier curves stratified by duration to diagnosis. Time to loss of ambulation in ALS patients, stratified by the median duration to diagnosis (11 months). The dotted line indicates shorter diagnostic delay (≤ median), and the solid line indicates longer delay (> median). Log‐rank test: *p* = 0.009.

**Figure 4:**
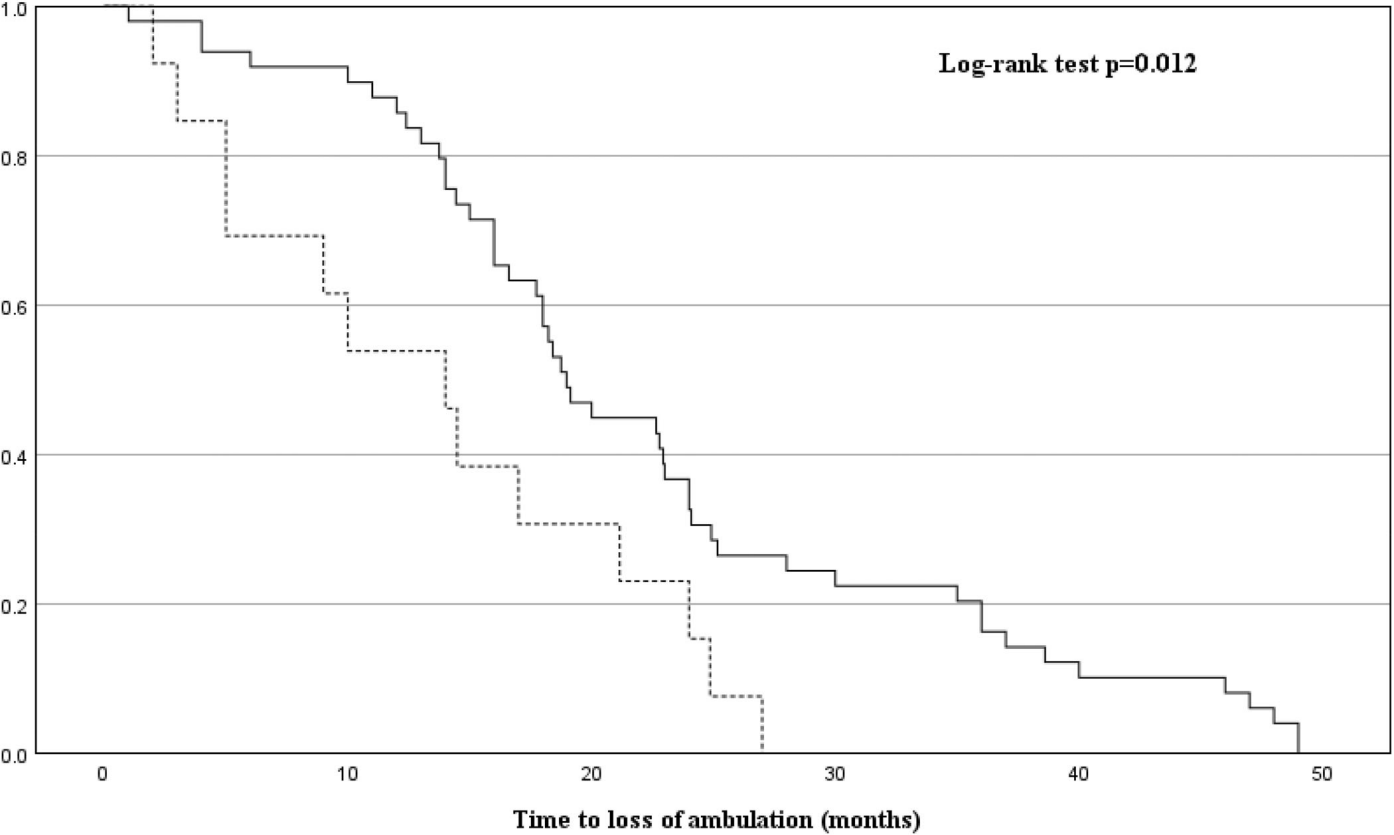
Kaplan–Meier survival curves comparing ALS patients with and without diabetes mellitus (DM). Time to loss of ambulation in ALS patients, stratified by the presence of diabetes mellitus (DM: yes vs no). The dotted line represents patients with DM, and the solid line represents those without DM. Log‐rank test: *p* = 0.012.

In the Cox proportional hazards regression analysis, adjusted for age at onset, sex, onset type, duration to diagnosis, ALS severity, diabetes mellitus, and %FVC, a significant association was found between longer duration to diagnosis and delayed loss of ambulation (HR = 0.94, 95% CI: 0.91–0.97, *p* < 0.001), as well as between the presence of DM and earlier loss of ambulation (HR = 3.30, 95% CI: 1.62–6.69, *p* < 0.001) (Table 3). These results are also visually summarized in the forest plot (Figure 2). Kaplan–Meier survival curves illustrated that patients with a longer duration to diagnosis (Figure 3) and those without DM (Figure 4) retained ambulation for a longer period.

**Table 3:** Cox proportional hazards model for factors associated with loss of ambulation in ALS patients.

Variable	Hazard Ratio (95% CI)	*p* value
Age at onset (years)	1.01 (0.99–1.04)	0.39
Sex (male vs female)	0.96 (0.55–1.67)	0.87
Onset type (Spinal vs Bulbar)	1.62 (0.84–3.12)	0.15
Duration to diagnosis (months)	0.94 (0.91–0.97)	< 0.001
ALS severity at diagnosis (grade)	1.27 (0.96–1.67)	0.10
Diabetes mellitus (yes vs no)	3.30 (1.62–6.69)	< 0.001
%FVC (%)	1.00 (0.98–1.01)	0.32

*Note:* Onset type: Spinal = 1, Bulbar = 0. ALS severity was assessed by clinical grade. %FVC = percent predicted forced vital capacity.

Abbreviations: CI, confidence interval; HR, hazard ratio.

To examine the robustness of these findings, a sensitivity analysis was conducted excluding patients with DM. The association between duration to diagnosis and time to loss of ambulation remained statistically significant (HR = 0.95, 95% CI: 0.92–0.98, *p* = 0.004) (Supporting Information Table 3).

## 4 Discussion

The study demonstrated that ALS patients with a shorter time to diagnosis and a history of DM were more likely to have an early deterioration in their ambulatory status.

In all ALS patients, the median time to loss of ambulation was 16.5 months, which was shorter than in previous studies [8, 22]. Over 65% of our study population was older than 65 years. Furthermore, half of the patients required assistance with activities of daily living at the time of diagnosis. The older the patient, the more rapid the disease progression and the more rapid the decline in motor function [23, 25]. Therefore, the time to loss of ambulation may have been shorter in our population.

Previous studies have reported that time from onset is associated with loss of ambulation [14] (Nakamura et al., 2013). It has also been shown that there is an independent association between length of time to diagnosis and delayed functional decline [26]. Herein, time to diagnosis had the highest impact on the time to loss of ambulation. Results reveal that time to diagnosis is associated with time to loss of ambulation, which is consistent with previous studies, as the rate of disease progression tends to be slower the longer the disease course [27]. Therefore, hearing the time to diagnosis may be useful in screening for ambulatory function.

The presence of DM in patients with ALS was associated with a shorter time to loss of ambulation. In our study, patients with a history of DM had a shorter time to loss of ambulation than those without DM. DM causes muscle weakness and atrophy associated with polyneuritis in the lower limbs [28, 29, 30]. Hyperglycemia increases oxidative stress and degenerates motor nerves [31, 32, 33, 34]. Additionally, DM and ALS patients have difficulty producing and regulating insulin‐like growth factor‐1 (IGF‐1), which promotes muscle hypertrophy and neuronal survival [35]. The effect of IGF‐1, which is common to DM and ALS patients, may have led to difficulties in muscle synthesis, which in turn may have led to ambulatory impairment.

The effect of DM on patients with ALS remains unclear. Conflicting data suggest protective and detrimental effects, and a previous study concluded that DM did not affect survival or disease progression [24]. However, it is possible that DM may have opposite effects on ALS in European and Asian populations owing to racial differences [36]. In addition, most previous studies have assessed risk factors associated with onset and survival; however, no reports have focused on the effect of DM on walking [24]. The presence of DM in people with ALS may worsen the disease when only lower‐limb function is considered [37].

Therefore, in ALS patients, the presence of DM may be associated with earlier loss of ambulation. Early implementation of walking aids and orthotic interventions is important for ALS patients with DM history. Regular physical exercise can increase IGF‐1 levels [38, 39, 40]; therefore, exploring exercise therapy and antioxidants after ALS onset may help extend ambulation duration.

The present study has a few limitations. First, the present study had a high age of onset and rapid progression of symptoms. The average age of onset in previous studies was 55–66 years [41], which may be difficult to generalize. Second, ALS is a rare disease with an incidence of 1–2.5 cases per 100,000 individuals [42], and the sample size of this study was relatively small, as it was conducted at a single institution. This limitation not only restricts the generalizability of our findings but also made meaningful statistical comparisons at specific time points (e.g., 1 year after symptom onset) challenging. Future studies should include larger cohorts from multiple institutions to improve the robustness of statistical analyzes and ensure broader applicability of the findings. Third, important clinical variables such as lower limb muscle strength (e.g., MRC score), nutritional status, and physical activity levels were not included in the analysis because these were not recorded in the medical records. Although BMI and grip strength were considered, they may not sufficiently capture lower limb function. Fourth, diabetes mellitus was assessed only as a binary variable (yes/no) based on medical records, and no information was available on glycemic control, disease duration, or diabetic complications. Finally, the variables included in the Cox regression model were selected based on clinical relevance and previous literature, but residual confounding due to unmeasured factors cannot be ruled out. As this was a retrospective observational study, no causal relationships can be established between the identified factors and loss of ambulation.

## 5 Conclusions

Our findings show that a timely diagnosis and the presence of DM are associated with earlier loss of ambulation in patients with ALS. Future research should include additional investigations in large samples and across a range of settings and disease stages to confirm the generalizability of these findings. Furthermore, it is necessary to test whether exercise therapy intervention from the time of diagnosis has an impact on reducing ambulation loss.

## Author Contributions

**Takahiro Miyashita:** writing – original draft, investigation, writing – review and editing, methodology, conceptualization, data curation, formal analysis. **Eiki Tsushima:** supervision, formal analysis, writing – original draft, writing – review and editing. **Hirofumi Ogihara:** supervision, formal analysis, writing—original draft, writing – review and editing.

## Ethics Statement

This study was performed after securing authorization from the Ethics Review Committee of Hirosaki University Graduate School of Medicine (Reference Number: 2021‐002) and the Ethics Review Committee of JA Nagano Koseiren Saku Central Hospital Clinical and Clinical Trial Center (Reference Number: 202005–07).

## Consent

The need for individual informed consent was waived by the Ethics Review Committee, and an opt‐out procedure was used in accordance with the ethical guidelines for medical and health research involving human subjects issued by the Ministry of Health, Labor and Welfare of Japan.

## Conflicts of Interest

The authors declare no conflicts of interest.

## Transparency Statement

The lead author Takahiro Miyashita affirms that this manuscript is an honest, accurate, and transparent account of the study being reported; that no important aspects of the study have been omitted; and that any discrepancies from the study as planned (and, if relevant, registered) have been explained.

## Supporting information


**SuppTable 1:** VariableCoding.

**SuppTable 2:** STROBE Checklist.

**SuppTable 3:** Sensitivity Analysis.

## Data Availability

The data that support the findings of this study are available from the corresponding author upon reasonable request.
