# Supplementary material for: Factors Influencing the Loss of Ambulation in Patients With Amyotrophic Lateral Sclerosis: A Retrospective Cohort Study
Source: Health Sci Rep. 2025 Sep 22;8(9):e71282. doi: 10.1002/hsr2.71282 (PMC12451060; doi:10.1002/hsr2.71282)
Supplement: Supplementary file 1 — SuppTable 1: VariableCoding. [file HSR2-8-e71282-s002.docx]

**Supplementary Table 1.**

Variable coding and definitions.

| **Variable** | **Type** | **Coding / Definition** |
| --- | --- | --- |
| Age at onset | Continuous (years) | Recorded as years |
| Sex | Binary | 0 = Female, 1 = Male |
| Onset type | Binary | 0 = Bulbar, 1 = Spinal |
| Duration to diagnosis | Continuous (months) | Number of months from onset to diagnosis |
| ALS severity | Ordinal (1–5) | 1 = mild (independent), 5 = severe (ventilator, tube feeding) |
| Diabetes mellitus | Binary | 0 = No, 1 = Yes |
| %FVC | Continuous (%) | Percent predicted forced vital capacity |
