# Supplementary material for: Factors Influencing the Loss of Ambulation in Patients With Amyotrophic Lateral Sclerosis: A Retrospective Cohort Study
Source: Health Sci Rep. 2025 Sep 22;8(9):e71282. doi: 10.1002/hsr2.71282 (PMC12451060; doi:10.1002/hsr2.71282)
Supplement: Supplementary file 2 — SuppTable 2: STROBE Checklist. [file HSR2-8-e71282-s001.docx]

Supplementary Table 2
STROBE Statement—checklist of items that should be included in reports of observational studies

|  | Item No. | Recommendation | Page  No. | Relevant text from manuscript |
| --- | --- | --- | --- | --- |
| **Title and abstract** | 1 | (*a*) Indicate the study’s design with a commonly used term in the title or the abstract | 1 | Title: '...a retrospective cohort study' clearly states the design. |
|  |  | (*b*) Provide in the abstract an informative and balanced summary of what was done and what was found | 2 | Abstract describes objectives, methods, and key findings. |
| Introduction | | | |  |
| Background/rationale | 2 | Explain the scientific background and rationale for the investigation being reported | 3 | Introduction explains ALS prognosis and importance of ambulation. |
| Objectives | 3 | State specific objectives, including any prespecified hypotheses | 3 | Objectives are clearly stated at the end of the Introduction. |
| Methods | | | |  |
| Study design | 4 | Present key elements of study design early in the paper | 3 | Methods section opens with study design. |
| Setting | 5 | Describe the setting, locations, and relevant dates, including periods of recruitment, exposure, follow-up, and data collection | 3 | Study conducted at Saku Central Hospital from May 2007 to Jan 2022. |
| Participants | 6 | (*a*) *Cohort study*—Give the eligibility criteria, and the sources and methods of selection of participants. Describe methods of follow-up  *Case-control study*—Give the eligibility criteria, and the sources and methods of case ascertainment and control selection. Give the rationale for the choice of cases and controls  *Cross-sectional study*—Give the eligibility criteria, and the sources and methods of selection of participants | 3 | Eligibility criteria and selection methods detailed under 'Patients'. |
|  |  | (*b*) *Cohort study*—For matched studies, give matching criteria and number of exposed and unexposed  *Case-control study*—For matched studies, give matching criteria and the number of controls per case | N/A |  |
| Variables | 7 | Clearly define all outcomes, exposures, predictors, potential confounders, and effect modifiers. Give diagnostic criteria, if applicable | 3-5 | Outcomes and covariates (e.g., DM, ALS severity) defined. |
| Data sources/ measurement | 8* | For each variable of interest, give sources of data and details of methods of assessment (measurement). Describe comparability of assessment methods if there is more than one group | *4-5* | *Data sources: electronic records, clinical testing; methods described.* |
| Bias | 9 | Describe any efforts to address potential sources of bias | 13-14 | Limitations include potential for unmeasured confounding. |
| Study size | 10 | Explain how the study size was arrived at | 3 | No formal sample size calculation: all eligible cases included. |

Continued on next page

| Quantitative variables | 11 | Explain how quantitative variables were handled in the analyses. If applicable, describe which groupings were chosen and why | 5-6 | Statistical section describes handling of variables. |
| --- | --- | --- | --- | --- |
| Statistical methods | 12 | (*a*) Describe all statistical methods, including those used to control for confounding | 5 | Cox regression model, variable selection and adjustments described. |
|  |  | (*b*) Describe any methods used to examine subgroups and interactions | N/A |  |
|  |  | (*c*) Explain how missing data were addressed | N/A |  |
|  |  | (*d*) *Cohort study*—If applicable, explain how loss to follow-up was addressed  *Case-control study*—If applicable, explain how matching of cases and controls was addressed  *Cross-sectional study*—If applicable, describe analytical methods taking account of sampling strategy | N/A |  |
|  |  | (*e*) Describe any sensitivity analyses | 9 | Sensitivity analysis excluding DM patients is reported. |
| Results | | | | |
| Participants | 13* | (a) Report numbers of individuals at each stage of study—eg numbers potentially eligible, examined for eligibility, confirmed eligible, included in the study, completing follow-up, and analysed | 3-4 | Flowchart (Fig.1) and text report eligible/included participants. |
|  |  | (b) Give reasons for non-participation at each stage | N/A |  |
|  |  | (c) Consider use of a flow diagram | 4 | Flowchart included (Fig.1). |
| Descriptive data | 14* | (a) Give characteristics of study participants (eg demographic, clinical, social) and information on exposures and potential confounders | 6-7 | Table 1: baseline and clinical characteristics. |
|  |  | (b) Indicate number of participants with missing data for each variable of interest | N/A |  |
|  |  | (c) *Cohort study*—Summarise follow-up time (eg, average and total amount) | 6 | Follow-up period: median time to loss of ambulation reported. |
| Outcome data | 15* | *Cohort study*—Report numbers of outcome events or summary measures over time | *6* | All patients lost ambulation; median and IQR provided. |
|  |  | *Case-control study—*Report numbers in each exposure category, or summary measures of exposure |  |  |
|  |  | *Cross-sectional study—*Report numbers of outcome events or summary measures |  |  |
| Main results | 16 | (*a*) Give unadjusted estimates and, if applicable, confounder-adjusted estimates and their precision (eg, 95% confidence interval). Make clear which confounders were adjusted for and why they were included | 9 | Table 3: adjusted hazard ratios and 95% CI; confounders listed. |
|  |  | (*b*) Report category boundaries when continuous variables were categorized | N/A |  |
|  |  | (*c*) If relevant, consider translating estimates of relative risk into absolute risk for a meaningful time period | N/A |  |

Continued on next page

| Other analyses | 17 | Report other analyses done—eg analyses of subgroups and interactions, and sensitivity analyses | 9 | Sensitivity analysis described in supplementary table. |
| --- | --- | --- | --- | --- |
| Discussion | | | | |
| Key results | 18 | Summarise key results with reference to study objectives | 12 | First paragraph of Discussion restates main findings. |
| Limitations | 19 | Discuss limitations of the study, taking into account sources of potential bias or imprecision. Discuss both direction and magnitude of any potential bias | 13-14 | Limitations discussed in detail (e.g., small sample, generalizability). |
| Interpretation | 20 | Give a cautious overall interpretation of results considering objectives, limitations, multiplicity of analyses, results from similar studies, and other relevant evidence | 12-14 | Interpretation considers other studies, mechanisms, and limitations. |
| Generalisability | 21 | Discuss the generalisability (external validity) of the study results | 12-14 | Generalisability discussed in context of sample size and age. |
| Other information | |  | | |
| Funding | 22 | Give the source of funding and the role of the funders for the present study and, if applicable, for the original study on which the present article is based | N/A |  |

*Give information separately for cases and controls in case-control studies and, if applicable, for exposed and unexposed groups in cohort and cross-sectional studies.

**Note:** An Explanation and Elaboration article discusses each checklist item and gives methodological background and published examples of transparent reporting. The STROBE checklist is best used in conjunction with this article (freely available on the Web sites of PLoS Medicine at http://www.plosmedicine.org/, Annals of Internal Medicine at http://www.annals.org/, and Epidemiology at http://www.epidem.com/). Information on the STROBE Initiative is available at www.strobe-statement.org.
