# Supplementary material for: Factors Influencing the Loss of Ambulation in Patients With Amyotrophic Lateral Sclerosis: A Retrospective Cohort Study
Source: Health Sci Rep. 2025 Sep 22;8(9):e71282. doi: 10.1002/hsr2.71282 (PMC12451060; doi:10.1002/hsr2.71282)
Supplement: Supplementary file 3 — SuppTable 3: Sensitivity Analysis. [file HSR2-8-e71282-s003.docx]

**Supplementary Table 3.**

| **Variable** | **Hazard Ratio (95% CI)** | ***p* value** |  |
| --- | --- | --- | --- |
| Age at onset (years) | 1.01 (0.98–1.04) | 0.40 |  |
| Sex (male vs female) | 0.84 (0.45–1.56) | 0.57 |  |
| Onset type (Spinal vs Bulbar) | 1.48 (0.69–3.19) | 0.31 |  |
| Duration to diagnosis (months) | 0.95 (0.92–0.98) | 0.004 |  |
| ALS severity (grade) | 1.11 (0.80–1.53) | 0.54 |  |
| %FVC (%) | 0.995 (0.98–1.01) | 0.38 |  |
| HR = hazard ratio; CI = confidence interval. Onset type: Spinal = 1, Bulbar = 0. ALS severity was assessed by clinical grade. %FVC = percent predicted forced vital capacity. | | |  |
|  |  |  |  |
|  |  |  |  |

Factors associated with loss of ambulation in ALS (DM-excluded Cox model).
